# Cigarette Smoke Extract and Nicotine Evoke Similar Interoceptive Effects in a Pavlovian Occasion Setting Task in Male and Female Sprague–Dawley Rats

**DOI:** 10.1093/ntr/ntaf219

**Published:** 2025-10-29

**Authors:** Anita Sikic, Davin R Peart, Mckenna A Williams, Avery R Cameron, Jessica M Karlovcec, Brandon W Florek, Jude A Frie, Jibran Y Khokhar, Rick A Bevins, Jennifer E Murray

**Affiliations:** Department of Psychology, University of Guelph, Guelph, ON, Canada; Collaborative Neurosciences Graduate Program, University of Guelph, Guelph, ON, Canada; Department of Psychology, University of Guelph, Guelph, ON, Canada; Collaborative Neurosciences Graduate Program, University of Guelph, Guelph, ON, Canada; Department of Molecular and Cellular Biology, University of Guelph, Guelph, ON, Canada; Department of Biomedical Sciences, University of Guelph, ON, Canada; Department of Molecular and Cellular Biology, University of Guelph, Guelph, ON, Canada; Department of Psychology, University of Guelph, Guelph, ON, Canada; Collaborative Neurosciences Graduate Program, University of Guelph, Guelph, ON, Canada; Department of Biomedical Sciences, University of Guelph, ON, Canada; Department of Anatomy and Cell Biology, University of Western Ontario, London, ON, Canada; Department of Psychology, University of Nebraska – Lincoln, Lincoln, NE, United States; Department of Psychology, University of Guelph, Guelph, ON, Canada; Collaborative Neurosciences Graduate Program, University of Guelph, Guelph, ON, Canada

## Abstract

**Introduction:**

Although nicotine is the primary component of interest in tobacco, the other ~9500 constituents in tobacco are thought to interact with nicotine to contribute to the pharmacological effects relevant to tobacco use disorder. These other tobacco constituents may also contribute to providing an interoceptive stimulus unique from that of nicotine alone. Using a Pavlovian drug discrimination task, we assessed whether rats could discriminate between nicotine and cigarette smoke extract (CSE) of the same nicotine concentration (0.2 mg/kg) based on the presence of constituent chemicals.

**Methods:**

Rats were assigned to one of three training conditions in which intermixed daily injections were administered before chamber placement. The interoceptive stimulus elicited by the injected compound would set the occasion on which a light conditioned stimulus would, or would not, be followed by sucrose. Increased dipper entries during the conditioned stimulus indicate greater anticipation of impending sucrose. Groups included (1) nicotine versus vehicle with nicotine signaling sucrose, (2) CSE versus vehicle with CSE signaling sucrose, and (3) CSE versus nicotine with CSE signaling sucrose. This final group determined whether rats could discriminate based on other tobacco constituents.

**Results:**

Subjects readily discriminated between nicotine and vehicle and between CSE and vehicle within ~20 training sessions; however, they were unable to discriminate between CSE and nicotine after 72 sessions.

**Conclusions:**

Our results confirm that CSE is a successful Pavlovian discriminative stimulus and add to previous nicotine literature. Interestingly, we demonstrate that CSE and nicotine do not create distinct interoceptive environments under current training conditions.

**Implications:**

Nicotine has long been used as a proxy for tobacco in non-human animal studies. However, there has always been an undercurrent regarding the appropriateness of this approach given the myriad constituents in tobacco. Though generalization to other behavioral preparations must be done with extreme caution, the current findings suggest that, at least in some capacity, there is overlap in the stimulus characteristics of nicotine and CSE.

## Introduction

Despite increased awareness of the adverse effects of smoking cigarettes, tobacco use remains the leading risk factor for premature mortality today. On a global scale, ~1.3 billion individuals smoke cigarettes, contributing to an estimated 8 million premature deaths per year, rendering the tobacco epidemic one of the greatest public health concerns in history.^1^ In Canada, their use in the general population remains high at 10.9% for individuals over the age of 15.^2^ Beyond its well-known addictive properties, cigarette smoke contains a vast number of constituents. Unsurprisingly, the number of these identified constituents has increased over the past few decades. For example, a review from 1959 suggested the presence of ~400 constituents in tobacco and its smoke,^3^ whereas the current estimate lies at ~9500.^4^ Therefore, it is possible that these estimations are still not exhaustive and will continue to increase alongside the advancement of analytical techniques.

Nicotine is the primary alkaloid found in tobacco and is generally accepted as the component responsible for its addictive properties, given its action on reward and reinforcement centers of the brain. Although nicotine is the key component of interest related to tobacco use, the other constituents in cigarette smoke are thought to modulate the effects of nicotine, contributing to the pharmacological impacts relevant to cigarettes’ addictive properties. Constituents, including acetaldehydes, monoamine oxidase (MAO) inhibitors, and minor tobacco alkaloids, have been found to mimic and/or enhance nicotine.^5–8^ However, despite awareness of many other constituents within cigarette smoke, most preclinical studies have investigated the mechanisms of nicotine alone, thereby discounting the possible influence of non-nicotine constituents on the brain and behavior. Therefore, the use of nicotine alone as a proxy for cigarettes is limited in its real-world translatability, which is slowly becoming evident in the literature. For example, a study investigating the effects of five minor alkaloids found in cigarette smoke (anabasine, nornicotine, anatabine, cotinine, and myosmine) found that rats self-administered nicotine plus these alkaloids significantly more than nicotine on its own, and exhibited higher locomotor activity, together suggesting that these constituents may facilitate smoking behavior.^6^ Furthermore, for a comprehensive understanding of tobacco-use disorder and thereby its treatment, preclinical behavioral research should reflect reality by also taking all the constituents of tobacco smoke into account. This has begun to be the case somewhat recently, with behavioral researchers incorporating the use of whole cigarette smoke extract (CSE) in animal models.^6^^,^^9–12^

Occasion setting offers an important perspective within drug discrimination tasks and permits the study of the extent to which internal stimuli are perceptible and if their associations with environmental stimuli can be learned. In this higher-order Pavlovian task, an occasion setter (OS), such as a drug, indirectly modulates the association between a conditioned stimulus (CS) and its consequential outcome, the unconditioned stimulus (US).^13^^,^^14^ Thus, the interoceptive effects of a drug are used to disambiguate a CS-US association, such as a light (as in the present study) or noise presentation, and a subsequent sucrose reward.^15^ This task involves feature positive (FP) or feature negative (FN) training in which the OS can initiate or inhibit a conditioned response (eg sucrose/reward-seeking) to a CS presentation, respectively. In FP training, for example, this is evidenced by an animal’s behavior such that it will actively seek the US during a CS presentation on trials where the drug stimulus is present and inhibit this reward-seeking behavior when the drug stimulus is absent. Several drugs of abuse have been established as successful OSs, including caffeine,^16^ methadone,^17^ cocaine,^18^ amphetamine and chlordiazepoxide,^19^ and morphine.^20–23^ Regarding nicotine, previous occasion-setting literature has established it as a successful FP^19^^,^^24^^,^^25^ and FN^26–29^ OS in rats. To date, no studies have been done so with CSE, nor have any investigated whether animals can discriminate between the two substances without prior training on nicotine (see^30^). If animals are indeed able to discriminate nicotine from CSE at equivalent nicotine concentrations, this would provide steadfast evidence that nicotine alone may not be an accurate proxy for tobacco smoke.

The aims of this study were 3-fold. First, to replicate the prior literature on nicotine as a FP OS. Second, to contribute novel findings on the ability for CSE to act as an OS. Finally, and of primary interest, to determine whether rats can discriminate nicotine from CSE at equal nicotine concentrations, essentially relying on the non-nicotine cigarette smoke constituents to guide the behavior.

## Methodology

### Materials

#### Subjects

Sixty Sprague–Dawley rats (30 males and 30 females; Charles River Lab, Kingston, NY, United States) were housed individually in standard opaque cages (polycarbonate; 50.5 × 48.5 × 20 cm) with standard bedding, one paper cup, and a plastic toy bone. Following 4 days of acclimation and then handling, animals were weighed daily and underwent 3 days of pre-training injections: nicotine (0.2 mg/kg), CSE (containing 0.2 mg/kg nicotine), and 1 day of the vehicle solution (see “Drug Preparation” for details). This was done to attenuate the initial locomotor suppressant effects of nicotine^31^ and ensure no adverse side effects occurred from the CSE. Animals were provided with water and food (Envigo, Madison, Wisconsin, Rodent Diet, 14% protein) *ad libitum* until the day before training commenced, when they were transitioned to food restriction (15 g for females; 20 g for males, 18% protein) to maintain healthy weights (~90% of free-fed body weight) throughout the experiment. Food was provided following each experimental session. All procedures were approved by the University of Guelph Animal Care Committee in accordance with the Canadian Council on Animal Care guidelines.

#### Drug Preparation

Concentrated CSE in dimethyl sulfoxide (DMSO) was ordered from Enthalpy Analytical (Richmond, VA, United States). Enthalpy provided the concentrated CSE in 1 mL aliquots (stored in -80°C until preparation) with 2 mg nicotine/mL after they collected the smoke on a filter and extracted it with DMSO to ensure capture of both hydrophobic and hydrophilic constituents. Quality assurance and nicotine concentration were confirmed by Enthalpy. This was then diluted in saline to achieve a nicotine concentration of 0.2 mg/mL; the ratio of DMSO to saline was 3:22 mL. Nicotine ditartrate dihydrate (Fisher Scientific, Ottawa, ON, CAN) was dissolved in equivalent concentrations of saline and DMSO (Sigma-Aldrich, MO, USA) to achieve a concentration of 0.2 mg/mL.^32–34^ The vehicle acted as a control and was prepared by combining saline and DMSO at exact concentrations to the nicotine and CSE solutions. The pH for all solutions was adjusted using 0.1 M NaOH to 7.2–7.4. Injections were administered subcutaneously 5 minutes before each session, and animals were weighed daily to determine their volume of either CSE, nicotine, or vehicle solution administered at 1 mL/kg.

#### Apparatus

The present study used 10 standard conditioning chambers for training (ENV-018, Med Associates, Georgia, VT; 30.5 L × 24.1 W × 21.0H cm), each of which was inside of a sound and light-attenuated enclosure. The sidewalls, trays, and grid floors of the chambers were made of aluminum; the ceiling, as well as the front and back walls, were made of transparent polycarbonate. The sucrose dipper receptacle was located on the right-hand side of the chamber, where rats could receive access to sucrose solution (26% w/v). A motor-operated dipper arm located just outside of the receptacle provided 4 seconds of access to 0.1 mL of sucrose solution within the receptacle, when appropriate. The cue light was located at the top left-hand side of the chamber, and a photobeam located inside the dipper receptacle measured head entries or “nose pokes”. An emitter-detector unit crossing the chamber 6 cm above the floor grid and 5.5 cm from the right wall tracked general locomotor activity throughout sessions. Stimulus outputs were controlled by a MedAssociates interface, and inputs were recorded by MedAssociates software.

### Behavioral Procedures

#### Magazine Training

All rats first underwent 3 days of magazine training to familiarize the rats with the chambers used for training and to teach them how to retrieve sucrose. This training consisted of ~50-minute sessions, in which they were required to access 4-second deliveries of 0.1 mL of 26% sucrose solution 51 times across the session for it to terminate.

#### Experimental Group Assignment

Subjects were randomly assigned to three groups as follows: nicotine versus vehicle (NIC/VEH; *n* = 19; 10 M, 9F) in which NIC was FP and VEH was FN, CSE versus vehicle (CSE/VEH; *n* = 20; 10 M, 10F) in which CSE was FP and VEH was FN, and CSE versus nicotine (CSE/NIC; *n* = 20; 10 M, 10F) in which CSE was FP and NIC was FN. Note that one female was excluded from the NIC/VEH group due to an accidental injury, which was unrelated to the study methods. Within each group, subjects had intermixed sessions of receiving the FP and the FN injections and programs; sessions were random and counterbalanced with the restriction that no more than two of a particular training session occurred in succession.

#### Drug Discrimination (Acquisition) Sessions

Each rat received an injection of one of their allocated solutions 5 minutes before the start of the daily session.^29^^,^^32^^,^^33^^,^^35^ All sessions were 20 minutes long and had eight 15-second presentations of the light cue (the CS). We used four versions of the program that varied by initial blackout duration (range: 120–240 seconds) and intertrial interval (range: 45–165 seconds). Following the offset of each light presentation on FP sessions, 4-second access to 0.1 mL of 26% sucrose (the US) was provided. Sucrose was withheld following the light presentations on FN sessions. This training occurred across 72 sessions. After the 36^th^ paired session (meaning 36 FP and 36 FN sessions), only the CSE/NIC group was transitioned from NIC to VEH solution as the FN, keeping CSE the FP, while the remaining two groups continued as normal; this continued until 46 paired sessions.

#### Dependent Measures

Fifteen seconds before the onset of the first light cue (CS) presentation, nose pokes into the dipper entryway were recorded by a photobeam. This measure of the number of dipper entries during the 15 seconds before the first light onset is referred to as the first pre-CS entries. This is used as a measure of their baseline reward-seeking behavior. During activation of the first 15-second light cue, nose pokes were again recorded, and these are referred to as the first-CS entries. First-CS entries, therefore, represent anticipatory reward-seeking behavior elicited by the CS-US pairing and should be higher on FP sessions than FN sessions if the animal has learned the correct discrimination (see Supplementary Material for results pertaining to these two measures). Subtracting the first pre-CS entries from the first-CS entries yields the primary measure of interest, which is the first dipper entry difference score. Therefore, behavior before and during only the first CS presentation is used rather than the mean across the entire session. This allows measurement of the anticipatory behavior of the animal that is evoked by the CS through the guidance of the OS without confound from within-session sucrose presence or absence. Therefore, a positive difference score indicates that the light cue evoked an anticipation of the sucrose reward, while a score of zero indicates no such change in behavior.^19^^,^^22^^,^^23^^,^^25^ Total locomotor activity is also measured via beam breaks during each session.

### Data Analyses

A mixed three-way analysis of variance, ANOVA [2 (Sex: male, female) × 2 (Drug: FP, FN) × 46 (Session: 1–46); mANOVA] was conducted first for each group. Each group was then stratified by sex and analyzed by two-way repeated measures ANOVAs [2 (Drug: FP, FN) × 46 (Session: 1–46); rmANOVA]. Where appropriate, significant effects (*p* < .05) were analyzed using Fisher’s least significant difference (LSDmmd) post-hoc tests. Given that such post-hoc tests are not corrected for multiple comparisons, our reporting of these comparisons should be interpreted with caution. Data files and Enthalpy quality assurance documents for the CSE are available in the Open Science Framework data repository (https://osf.io/92ncx/; DOI: http://dx.doi.org/10.17605/OSF.IO/92NCX).

## Results

### Nicotine Feature Positive with Vehicle Feature Negative (NIC/VEH)

#### First Dipper Entry Difference Score

**Figure 1:**
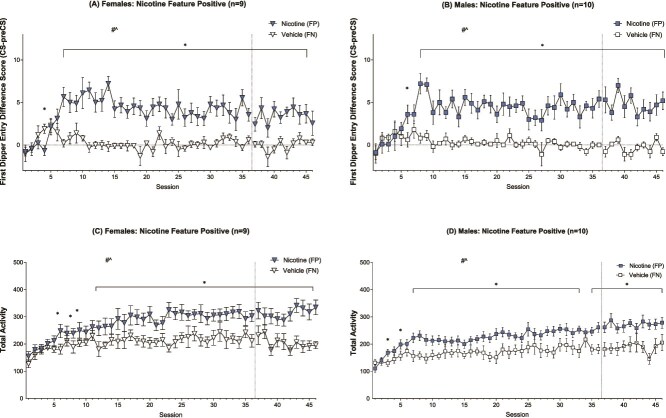
Nicotine (NIC) feature positive with vehicle (VEH) feature negative first dipper entry difference score means (±SEM) for female (A) and male (B) rats and total locomotor activity means for female (C) and male (D) rats in the NIC/VEH groups across 46 feature positive and 46 feature negative paired sessions. The # denotes a significant main effect of drug, and ^ denotes a significant main effect of session from a two-way within-subjects ANOVA. The ^*^ denotes a significant difference between means on those respective paired sessions (*p* < .05).

Male and female rats similarly acquired the discrimination between NIC (FP) and VEH (FN) solutions. The mANOVA indicated a significant Drug^*^Session interaction [F(45,765) = 4.698, *p* < .001, η_p_^2^ = .217], a main effect of Drug [F(1,17) = 92.299, *p* < .001, η_p_^2^ = .844] and a main effect of session [F(45,765) = 3.630, *p* < .001, η_p_^2^ = .176]. There were no significant Drug^*^Sex, Session^*^Sex, or Drug^*^Session^*^Sex interactions (see Figure 1A and B) and no main effect of Sex.

The rmANOVA for the females indicated a significant Drug^*^Session interaction [F(45,360) = 2.916, *p* < .001, η_p_^2^ = .267], a main effect of Drug [(F(1,8) = 49.936, *p* < .001, η_p_^2^ = .862], and a main effect of Session [F(45,360) = 2.566, *p* < .001, η_p_^2^ = .243; see Figure 1A]. Post-hoc analyses on the Drug^*^Session interaction indicated that females discriminated between NIC and VEH on paired session 4 and again on sessions 7-45 (LSDmmd = 2.254). In males, the rmANOVA indicated a significant Drug^*^Session interaction [F(45,405) = 3.125, *p* < .001, η_p_^2^ = .258], main effect of Drug [F(1,9) = 45.306, *p* < .001, η_p_^2^ = .834], and main effect of Session [F(45,405) = 2.324, *p* < .001, η_p_^2^ = .205; see Figure 1B]. Post-hoc tests indicated discrimination on paired sessions 6 and 8–46 (LSDmmd = 2.062).

#### Total Activity

The mANOVA indicated a significant main effect of Drug [F(1,17) = 48.747, *p* < .001, η_p_^2^ = .741], main effect of Session [F(45,765) = 11.104, *p* < .001, η_p_^2^ = .395], and a Drug^*^Session interaction [F(45,765) = 3.373, *p* < .001, η_p_^2^ = .166] on general locomotor activity throughout trials. There were no Drug^*^Sex, Session^*^Sex, or Drug^*^Session^*^Sex interactions (see Figure 1C and D), and no main effect of Sex.

Females showed a significant main effect of Drug [F(1,8) = 22.913, *p* = .001, η_p_^2^ = .741], main effect of Session [F(45,360) = 4.867, *p* < .001, η_p_^2^ = .378], and a Drug^*^Session interaction [F(45,360) = 2.340, *p* < .001, η_p_^2^ = .226; see Figure 1C]. Post-hoc tests indicated significantly greater activity on FP than FN trials for sessions 6, 8–9, and 12–46 (LSDmmd = 44.894). In males, the rmANOVA also yielded a significant main effect of Drug [F(1,9) = 26.308, *p* < .001, η_p_^2^ = .745], a main effect of Session [F(45,405) = 7.964, *p* < .001, η_p_^2^ = .469], and a Drug^*^Session interaction [F(45,405) = 2.096, *p* < .001, η_p_^2^ = .189; see Figure 1D]. Follow-up tests indicated greater activity on FP trials for sessions 3, 5, 7–33, and 35–46 (LSDmmd = 34.011).

### CSE Feature Positive with Vehicle Feature Negative (CSE/VEH)

#### First Dipper Entry Difference Score

**Figure 2:**
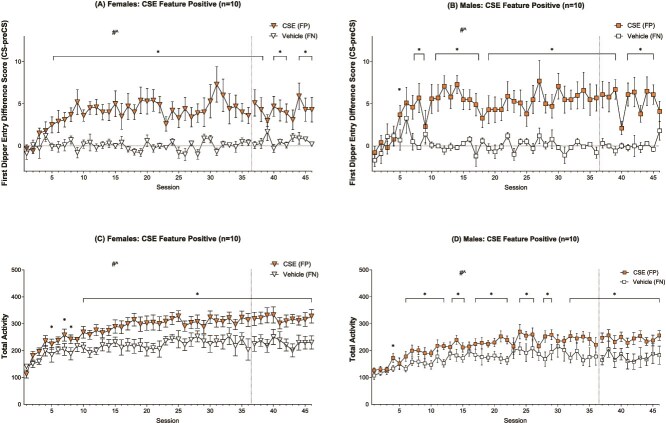
Cigarette smoke extract (CSE) feature positive with vehicle (VEH) feature negative first dipper entry difference score means (±SEM) for female (A) and male (B) rats, and total locomotor activity means for female (C) and male (D) rats in the CSE/VEH groups across 46 feature positive and 46 feature negative paired sessions. The # denotes a significant main effect of drug, and ^ denotes a significant main effect of session from a two-way within-subjects ANOVA. The ^*^ denotes a significant difference between means on those respective paired sessions (*p* < .05).

Both males and females similarly acquired the CSE (FP) versus VEH (FN) discrimination. The mANOVA yielded a significant main effect of Drug [F(1,18) = 72.990, *p* < .001, η_p_^2^ = .802], a main effect of Session [F(45,810) = 2.935, *p* < .001, η_p_^2^ = .140] and a Drug^*^Session interaction [F(45,810) = 3.019, *p* < .001, η_p_^2^ = .144] on the difference score (see Figure 2A and B), and no main effect of Sex.

Females demonstrated a significant main effect of Drug [F(1,9) = 30.510, *p* < .001, η_p_^2^ = .772], a main effect of session [F(45,405) = 1.672, *p* = .006, η_p_^2^ = .157], and a Drug^*^Session interaction [F(45,405) = 2.008, *p* < .001, η_p_^2^ = .182; see Figure 2A]. Post-hoc tests indicated significantly greater reward-seeking on FP trials for sessions 5–38, 40–42, and 44–46 (LSDmmd = 2.200). Males similarly demonstrated a significant main effect of Drug [F(1,9) = 42.828, *p* < .001, η_p_^2^ = .826], a main effect of Session [F(45,405) = 1.936, *p* < .001, η_p_^2^ = .177], and a Drug^*^Session interaction [F(45,405) = 2.311, *p* < .001, η_p_^2^ = .204; see Figure 2B]. Post-hoc tests in the males also demonstrated higher seeking on FP trials, but for sessions 5, 7–8, 10–17, 19–39, and 41–45 (LSDmmd = 2.751).

#### Total Activity

The mANOVA yielded a significant main effect of Drug [F(1,18) = 59.401, *p* < .001, η_p_^2^ = .767], main effect of Session [F(45,810) = 14.487, *p* < .001, η_p_^2^ = .446], and a Drug^*^Session interaction [F(45,810) = 2.690, *p* < .001, η_p_^2^ = .130] on general locomotor activity. There was also a main effect of Sex [F(1,18) = 5.417, *p* = .032, η_p_^2^ = .231] such that females displayed greater activity than males overall. There were no significant Drug^*^Sex, Session^*^Sex, or Drug^*^Session^*^Sex interactions (see Figure 2C and D).

Females demonstrated a significant main effect of Drug [F(1,9) = 40.781, *p* < .001, η_p_^2^ = .819], a main effect of Session [F(45,405) = 8.256, *p* < .001, η_p_^2^ = .478], and a Drug^*^Session interaction [F(45,405) = 1.932, *p* < .001, η_p_^2^ = .177; see Figure 2C]. Follow-up tests indicated significantly greater activity on CSE (FP) trials 5, 7–8, and 10–46 (LSDmmd = 38.063). Males demonstrated the same pattern of results with again a significant main effect of Drug [F(1,9) = 20.536, *p* = .001, η_p_^2^ = .695], Session [F(45,405) = 6.985, *p* < .001, η_p_^2^ = .437], and a Drug^*^Session interaction [F(45,405) = 1.585, *p* = .012, η_p_^2^ = .150; see Figure 2D]. Post-hoc testing revealed significantly greater activity on CSE (FP) trials for sessions 4, 6–12, 14–15, 17–22, 24–26, 28–29, and 32–46 (LSDmmd = 35.431).

### CSE Feature Positive with Nicotine Feature Negative (CSE/NIC)

#### First Dipper Entry Difference Score

**Figure 3:**
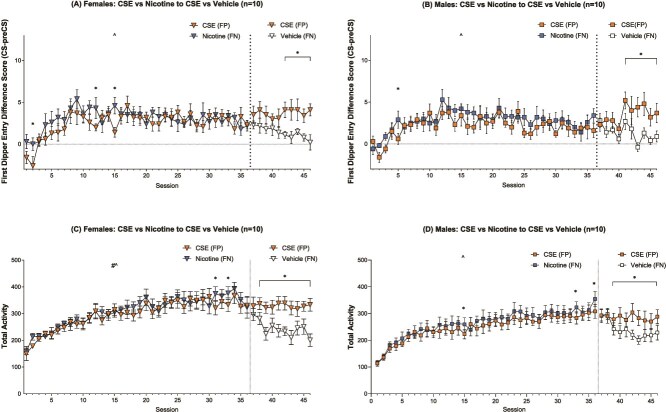
Cigarette smoke extract (CSE) feature positive with nicotine (NIC) feature negative first dipper entry difference score means (±SEM) for female (A) and male (B) rats, and total locomotor activity means for female (C) and male (D) rats in the CSE/NIC groups across 46 feature positive and 46 feature negative paired sessions. The # denotes a significant main effect of drug, and ^ denotes a significant main effect of session from a two-way within-subjects ANOVA. The ^*^ denotes a significant difference between means on those respective paired sessions (*p* < .05).

Male and female rats did not acquire the CSE/NIC discrimination. The mANOVA yielded a significant main effect of Session [F(45,810) = 4.879, *p* < .001, η_p_^2^ = .213] and a significant Drug^*^Session interaction [F(45,810) = 3.432, *p* < .001, η_p_^2^ = .160], but no significant main effects of Drug or Sex, or any Drug^*^Sex, Session^*^Sex, or Drug^*^Session^*^Sex interactions on the difference score (see Figure 3A and B).

Females demonstrated a significant main effect of Session [F(45,405) = 3.109, *p* < .001, η_p_^2^ = .257] and a Drug^*^Session interaction [F(45,405) = 2.407, *p* < .001, η_p_^2^ = .211; see Figure 3A]. This experimental group underwent the contingency shift wherein they were transitioned from NIC to VEH as the FN for sessions 37–46. Post-hoc tests on the interaction revealed significantly greater seeking on CSE (FP) trials on sessions 2, 12, and 15 (pre-shift), as well as 42–46 (post-shift; LSDmmd = 2.046). The males demonstrated a similar pattern with a main effect of Session [F(45,405) = 2.547, *p* < .001, η_p_^2^ = .221] and a Drug^*^Session interaction [F(45,405) = 1.953, *p* < .001, η_p_^2^ = .178; see Figure 3B]. Follow-up tests indicated significantly greater CSE (FP) sucrose-seeking on session 5 (pre-shift) and sessions 41–46 (post-shift; LSDmmd = 2.198).

#### Total Activity

The mANOVA on general locomotor activity yielded a significant main effect of Session [F(45,810) = 24.920, *p* < .001, η_p_^2^ = .581], a Drug^*^Sex interaction [F(1,18) = 5.301, *p* = .033, η_p_^2^ = .228], and a Drug^*^Session interaction [F(45,810) = 7.539, *p* < .001, η_p_^2^ = .295]. There were no significant main effects of Drug or Sex, and no Session^*^Sex or Drug^*^Session^*^Sex interactions (see Figure 3C and D).

Females demonstrated a significant main effect of Drug [F(1,9) = 5.251, *p* = .048, η_p_^2^ = .368], a main effect of Session [F(45,405) = 11.717, *p* < .001, η_p_^2^ = .566], and a Drug^*^Session interaction [F(45,405) = 5.346, *p* < .001, η_p_^2^ = .373; see Figure 3C]. Post-hoc testing revealed significantly greater locomotion of CSE (FP) trials for sessions 31 and 33 (pre-shift) and 38–46 (post-shift; LSDmmd = 38.495). The males demonstrated a significant main effect of Session [F(45,405) = 14.956, *p* < .001, η_p_^2^ = .624] and a Drug^*^Session interaction [F(45,405) = 3.117, *p* < .001, η_p_^2^ = .257], but no main effect of Drug (see Figure 3D). Follow-up tests indicated significantly higher CSE (FP) activity on sessions 15, 33, and 36 (pre-shift) and 39–46 (post-shift; LSDmmd = 34.894).

## Discussion

The primary purpose of the present study was to determine whether the interoceptive effects of the non-nicotine cigarette smoke constituents are sufficient to guide behavior in an appetitive drug discrimination task. NIC/VEH and CSE/VEH groups included as controls demonstrated that the stimulus effects of both nicotine and CSE successfully function as FP OSs in both male and female rats. Males and females in both groups reliably demonstrated goal-tracking behavior during the light presentation on FP trials when the CS-US association was active. They also learned to inhibit this behavior on FN trials before which they were exposed to vehicle solution, and the CS-US association was therefore inactive. These data demonstrate that these drugs functioned as OSs rather than conditioned stimuli; approach behavior was driven by modulation rather than induction of goal-tracking (see Supplemental Results). Thus, results are consistent with previous studies, which established nicotine as a FP OS^19^^,^^24^^,^^25^ and CS^31^^,^^35–37^ in rats. Furthermore, the present study contributes novel findings for the ability of whole-tobacco smoke extract to function as a FP OS.

These results also demonstrated that rats either could not discriminate between the interoceptive stimuli produced by nicotine administration alone and those produced by whole-tobacco smoke administration or could not use these stimuli to appropriately guide reward-seeking behavior in an occasion setting task at a moderate dose (eg^32^^,^^33^). This finding suggests that the non-nicotine constituents in CSE do not alone create a distinct interoceptive environment or modify the interoceptive environment produced by nicotine to an extent sufficient to alter the use of this environment to guide behavior. While the results of the present study initially appear at odds with reports suggesting that the addition of one or a few constituents enhances the reinforcing effects of nicotine,^5^^,^^6^^,^^38^ but see,^39^ which could involve modulation of the interoceptive stimuli evoked by the reinforcer, they appear consistent with those indicating that CSEs are equally or less reinforcing than nicotine alone.^9^^,^^11^^,^^12^ Interestingly, Smith et al.^39^ showed that a cocktail of five minor alkaloids (anabasine, nornicotine, cotinine, myosmine, and anatabine), two β-carbolines (harmane and norharmane), and acetaldehyde did not enhance nicotine self-administration in rats; however, they included the β-carbolines, which are reversible MAO inhibitors found in tobacco smoke. Irreversible MAO inhibition has been shown to increase nicotine SA in mice^40^ and rats,^7^ as such, the effect of CSE constituents on the behavioral effects of nicotine may depend on the MAO-inhibitory properties of the selected constituents. The role of MAO inhibition is complex and beyond the scope of this study, so we refer interested readers to Hogg^41^ for more information on this topic.

Given our finding that the complete combination of cigarette smoke constituents did not affect interoception differently than nicotine alone at our dose (0.2 mg/kg), it is plausible that the lack of effects of these constituents on nicotine reinforcement in SA studies is resultant of a similarity in the interoceptive stimuli produced by these reinforcers. However, this is somewhat inconsistent with results from Lee et al.^30^ in which male rats were trained on 0.4 mg/kg nicotine before generalization testing with various doses of nicotine alone and in CSE. They suggest that CSE and nicotine exhibited similar interoceptive cues generally, but lower doses of nicotine in CSE allowed for partial and full substitution of the training dose compared to nicotine alone. Coupled with a lower effective dose 50 (ED_50_) value for CSE (0.029 mg/kg) than nicotine (0.12 mg/kg), this finding suggests that the constituents enhance the potency or salience of nicotine ~4-fold.^30^ This may explain a lack of discrimination in the present study, as well as present a limitation, such that our dose exceeds the threshold of generalization, resulting in a ceiling effect. However, it is also possible that the nature of their task (two-lever operant discrimination) in combination with substitution testing created conditions under which animals could more easily discriminate the interoceptive cues elicited by the substances and doses. Additionally, while not another limitation per se, it would be valuable to include analyses of major constituents in all studies using whole extracts. This would allow for better understanding of whether differences in these concentrations result in differential effects across studies as the literature continues to grow. Finally, it is important to note that CSE is inconsistent between studies due to various possible methods of obtaining the substance. Consistent and translatable methods are imperative to a cohesive understanding of tobacco-use disorder and the development of potential treatments; thus, careful consideration should be given to the method of obtaining whole extracts in future studies.

## Supplementary Material

Nic_CSE_Supplementary_Material_Final_ntaf219

## Data Availability

Data files and Enthalpy quality assurance documents for the CSE are available in the Open Science Framework data repository (https://osf.io/92ncx/; DOI: http://dx.doi.org/10.17605/OSF.IO/92NCX).
