# Supplementary material for: Cigarette Smoke Extract and Nicotine Evoke Similar Interoceptive Effects in a Pavlovian Occasion Setting Task in Male and Female Sprague–Dawley Rats
Source: Nicotine Tob Res. 2025 Oct 29;28(5):783–90. doi: 10.1093/ntr/ntaf219 (PMC13101993; doi:10.1093/ntr/ntaf219)
Supplement: Nic_CSE_Supplementary_Material_Final_ntaf219 [file nic_cse_supplementary_material_final_ntaf219.docx]

Cigarette smoke extract and nicotine evoke similar interoceptive effects in a Pavlovian occasion setting task in male and female Sprague-Dawley rats

**Supplementary Material**

Anita Sikic^1,2^ MSc, Davin R. Peart^1,2^  MSc, Avery R. Cameron^3^ BSc, Jessica M. Karlovcec^5^ BSc, Mckenna A. Williams^5^ BSc, Brandon W. Florek^1^ BSc, Jude A. Frie^2,3^ PhD, Jibran Y. Khokhar^6^ PhD, Rick A. Bevins^4^ PhD, Jennifer E. Murray^1,2^ PhD

^1^Department of Psychology, University of Guelph, Guelph, ON, Canada

^2^Collaborative Neurosciences Graduate Program, University of Guelph, Guelph, ON, Canada

^3^Department of Biomedical Sciences, University of Guelph, ON, Canada

^4^Department of Psychology, University of Nebraska – Lincoln, Lincoln, NE, USA

^5^Department of Molecular and Cellular Biology, University of Guelph, Guelph, ON, Canada

^6^Department of Anatomy and Cell Biology, University of Western Ontario, ON, Canada

Corresponding author email: jmurr@uoguelph.ca

Corresponding author phone number: +1 519-824-4120

**Nicotine Feature Positive (NIC/VEH)**

*First Pre-CS Entries*

The mANOVA indicated a main effect of Drug [F(1,17)= 10.585, p=.005, η_p_^2^=.384], Session [F(45,765)=2.041, p<.001, η_p_^2^=.107], and Sex [F(1,17)= 6.977, p=.017, η_p_^2^=.291], but no Drug*Sex, Session*Sex, Drug*Session, or Drug*Session*Sex interactions (see Figure S1A and S1B).

Females demonstrated a significant main effect of Drug [F(1,8)=7.564, p=.025, η_p_^2^=.486] such that nose pokes into the receptacle were higher on NIC (FP) than VEH (FN) trials on average, but no significant main effect of Session or Drug*Session interaction (see Figure S1A). Males demonstrated a significant main effect of Session [F(45,405)=2.000, p<.001, η_p_^2^=.182] such that nose pokes into the receptacle decreased across sessions. There was no significant main effect of Drug and no Drug*Session interaction (see Figure S1B).

### *First CS Entries*

The mANOVA indicated a significant main effect of Drug [F(1,17)=109.967, p<.001, η_p_^2^=.866], main effect of Session [F(45,765)=3.257, p<.001, η_p_^2^=.161], and a Drug*Session interaction [F(45,765)=5.899, p<.001, η_p_^2^=.258] on first-CS entries. There were no significant Drug*Sex, Session*Sex, or Drug*Session*Sex interactions (see Figure S1C and S1D) and no main effect of Sex.

Females showed a significant main effect of Drug [F(1,8)=57.778, p<.001, η_p_^2^=.878], main effect of Session [F(45,360)=2.495, p<.001, η_p_^2^=.238], and a Drug*Session interaction [F(45,360)=3.984, p<.001, η_p_^2^=.332]. Post-hoc tests indicated significantly greater FP CS-entries on sessions 6-46 (LSDmmd=1.846; see Figure S1C). The males also demonstrated a significant main effect of Drug [F(1,9)=54.788, p<.001, η_p_^2^=.859], main effect of Session [F(45,405)=1.828, p=.001, η_p_^2^=.169] and a Drug*Session interaction [F(45,405)=3.292, p<.001, η_p_^2^=.268; see Figure S1D]. Follow-up tests again indicated significantly higher first CS-entries on FP trials for sessions 6-46 (LSDmmd=1.833).

**Figure S1.** **Nicotine Feature Positive with Vehicle Feature Negative**

First pre-CS entries means (±SEM) for female (A) and male (B) rats and first-CS means for female (C) and male (D) rats in the NIC/VEH groups across 46 FP and 46 FN paired sessions. The # denotes a significant main effect of drug and ^ denotes a significant main effect of session from a 2-way within-subjects ANOVA. The * denotes a significant difference between means on those respective paired sessions (p<.05).

## CSE Feature Positive (CSE/VEH)

### *First Pre-CS Entries*

The mANOVA yielded only a significant main effect of Session [F(45,810)=2.073, p<.001, η_p_^2^=.103]; no main effects of Drug or Sex, or any interactions were observed (see Figure S2A and S2B).

The rmANOVA in females yielded no significant main effects of Drug or of Session but did reveal a significant Drug*Session interaction [F(45,405)=1.606, p=.010, η_p_^2^=.151; see Figure S2A]. Follow-up post-hoc tests indicated significantly higher pre-CS entries on FP trials for sessions 3, 7-8, 14, 23, 26, 33, & 39 (LSDmmd=1.150). In contrast, the males demonstrated a significant main effect of Session [F(45,405)=1.559, p=.015, η_p_^2^=.148; see Figure S2B], such that first pre-CS entries decreased for both trial types across sessions, but no main effect of Drug and no Drug*Session interaction.

### *First CS Entries*

The mANOVA indicated a significant main effect of Drug [F(1,18)=71.003, p<.001, η_p_^2^=.798], main effect of Session [F(45,810)=2.400, p<.001, η_p_^2^=.118], and a Drug*Session interaction [F(45,810)=4.288, p<.001, η_p_^2^=.192]. There were no significant Drug*Sex, Session*Sex, or Drug*Session*Sex interactions (see Figure S2C and S2D), and no main effect of Sex.

The rmANOVA in females yielded a significant main effect of Drug [F(1,9)=30.190, p<.001, η_p_^2^=.770], main effect of Session [F(45,405)=1.423, p=.043, η_p_^2^=.137], and a Drug*Session interaction [F(45,405)=2.429, p<.001, η_p_^2^=.213; see Figure S2C]. Post-hoc tests revealed significantly greater CS-entries on FP trials for sessions 5-46 (LSDmmd=2.080). The males demonstrated the same pattern of results with a significant main effect of Drug [F(1,9)=41.231, p<.001, η_p_^2^=.821], main effect of Session [F(45,405)=1.798, p=.002, η_p_^2^=.166], and a Drug*Session interaction [F(45,405)=2.900, p<.001, η_p_^2^=.244; see Figure S2D]. Follow-up tests again revealed significantly greater CS-entries on FP trials but for sessions 7-39 and 41-46 (LSDmmd=2.496).

**Figure S2. Cigarette Smoke Extract Feature Positive with Vehicle Feature Negative**

First pre-CS entries means (±SEM) for female (A) and male (B) rats and first-CS means for female (C) and male (D) rats in the CSE/VEH groups across 46 FP and 46 FN paired sessions. The # denotes a significant main effect of drug and ^ denotes a significant main effect of session from a 2-way within-subjects ANOVA. The * denotes a significant difference between means on those respective paired sessions (p<.05).

## CSE Feature Positive (CSE/NIC)

### *First Pre-CS Entries*

The mANOVA on first pre-CS entries only yielded a significant main effect of Session [F(45,810)=2.813, p<.001, η_p_^2^=.135]; therefore, no significant main effects of Drug or Sex, or any Drug*Sex, Session*Sex, Drug*Session, or Drug*Session*Sex interactions were observed (see Figure S3A and S3B).

Both rmANOVAs similarly yielded only a significant main effect of Session [F(45,405)=2.272, p<.001, η_p_^2^=.202; see Figure S3A] in the females and in the males [F(45,405)=1.417, p=.045, η_p_^2^=.136; see Figure S3B] such that first pre-CS entries decreased over time.

### *First CS Entries*

The mANOVA on first CS entries demonstrated only a significant main effect of Session [F(45,810)=3.114, p<.001, η_p_^2^=.147] and a Drug*Session interaction [F(45,810)=4.214, p<.001, η_p_^2^=.190]. There were no significant main effects of Drug or Sex, and no Drug*Sex, Session*Sex, or Drug*Session*Sex interactions (see Figure S3C and S3D).

Females demonstrated a significant main effect of Session [F(45,405)=1.877, p<.001, η_p_^2^=.173] and a Drug*Session interaction [F(45,405)=2.626, p<.001, η_p_^2^=.226; see Figure S3C]. Follow-up tests indicated significantly greater FP reward-seeking on sessions 4, 12, 15, 39 (pre-shift) and 41-46 (post-shift; LSDmmd=1.829). The males demonstrated the same pattern of results with a significant main effect of Session [F(45,405)=1.918, p<.001, η_p_^2^=.176] and a Drug*Session interaction [F(45,405)=2.609, p<.001, η_p_^2^=.225; see Figure S3D] with significantly greater FP seeking on sessions 5, 14 and 36 (pre-shift) and 41-46 (post-shift; LSDmmd=2.049).

**Figure S3. Cigarette Smoke Extract Feature Positive with Nicotine Feature Negative**

First pre-CS entries means (±SEM) for female (A) and male (B) rats and first-CS means for female (C) and male (D) rats in the CSE/NIC groups across 46 FP and 46 FN paired sessions. The # denotes a significant main effect of drug and ^ denotes a significant main effect of session from a 2-way within-subjects ANOVA. The * denotes a significant difference between means on those respective paired sessions (p<.05).
